# Perception towards Online Classes during COVID-19 among MBBS and BDS Students in a Medical College of Nepal: A Descriptive Crosssectional Study

**DOI:** 10.31729/jnma.5348

**Published:** 2021-03-31

**Authors:** Nitasha Sharma, Chet Kant Bhusal, Sandip Subedi, Rajeshwar Reddy Kasarla

**Affiliations:** 1 Department of Anatomy, Universal College of Medical Sciences and Teaching Hospital, Bhairahawa, Rupandehi, Nepal; 2 Department of Community Medicine, Universal College of Medical Sciences and Teaching Hospital, Bhairahawa, Rupandehi, Nepal; 3 Department of Psychiatry, Universal College of Medical Sciences and Teaching Hospital, Bhairahawa, Rupandehi, Nepal; 4 Department of Microbiology, Universal College of Medical Sciences and Teaching Hospital, Bhairahawa, Rupandehi, Nepal

**Keywords:** *face-to-face teaching*, *medical students*, *online learning*

## Abstract

**Introduction:**

Sudden outbreak of COVID-19 pandemic has affected the educational system worldwide, forced the medical colleges to close due to lock down, and disrupted the classroom face-to-face teaching process. As a result, medical colleges shifted to an online mode of teaching. The aim of this study is to find out the perception towards online classes during COVID-19 lockdown period among MBBS and BDS students at a medical college of Nepal.

**Methods:**

This was a descriptive cross-sectional study carried out at Universal College of Medical Sciences and Teaching Hospital among first and second year Bachelor in Medicine, Bachelor of Surgery and Bachelor in dental surgery students from 1st June 2020 to 30th August 2020. Ethical approval was taken from Institutional Review Committee of Universal College of Medical Sciences and Teaching Hospital (IRC UCMS, Ref: UCMS/IRC/025/20). Convenient sampling method was used. Semi-structured questionnaire was used. Statistical Package for Social Sciences 22 was used for analysis and frequency and percentage was calculated.

**Results:**

One hundred fifty six (73.93%) students were enjoying online learning only to some extent, 135 (63.98%) felt online class not equally effective as face-to-face teaching. The students had disturbance during online classes as internet disturbance 168 (79.60%), and electricity problem 47 (22.3%). Similarly, many students 155 (73.50%) felt external disturbance, headache 26 (12.3%), and eye strain 26 (12.3%).

**Conclusions:**

Most of the students suffered from disturbances during online classes probably because of internet and electricity problem. When compulsory to conduct online classes, students felt that not more than three online classes per day should be conducted to avoid eye strain and headache.

## INTRODUCTION

The global pandemic COVID-19 has made an impact on each and every field. Medical education is no exception to it, with lockdown and social distancing measures face to face classes are abandoned.^1^ This forced educational institutions to shift to an online mode of teaching-learning, which is an imperfect yet quick solution to the crises.^2^ As per guidelines of Ministry of health and education, Universal College of Medical Sciences (UCMS) also started online classes.^3^

Online learning is a relatively new phenomenon, currently both the Teachers and students are struggling with the idea of its implementation and adaptation respectively.^4^ In a developing country like Nepal, several technological, education/literacy background and socioeconomic challenges exist, which might act as a hindrance to the online learning process.^5,6^

The aim of this study is to the perception towards online classes and learning during COVID-19 lockdown period among MBBS and BDS students at a medical college of Nepal.

## METHODS

A descriptive cross-sectional study was conducted among 1^st^ and 2^nd^ year Bachelor of Medicine and Bachelor of Surgery (MBBS) and Bachelor of Dental Surgery (BDS) at Universal College of Medical Sciences, Bhairahawa, Nepal from 1^st^ June 2020 to 30^th^ August 2020.

Ethical approval was taken from Institutional Review Board of Universal college of Medical Sciences and Teaching Hospital, Bhairahawa, Rupandehi, Nepal (IRC UCMS, Ref: UCMS/IRC/025/20). The objectives of the study were briefed to respondents. Written informed consent was taken from the students and additional verbal consent was taken from the students' parents through telephone for those students who were below 18 years of age. The data was used only for this study purpose and confidentiality of the data has been maintained.

All the medical and dental students studying in 1^st^ and 2^nd^ year at UCMS Bhairahawa, Nepal and wiliness to participate in the study were included in the study. Similarly those who were not wiliness to participate and those who did not fill questionnaire completely until the study period were excluded from the study. Convenient sampling method was used. A total of 211 questionnaires were completely filled and included in the study.

The minimum sample size was estimated using the following formula:

$$\begin{array}{ll} \text{n} & ={\text{Z}}^{\text{2}}\times \text{p}\times \text{q}/{\text{e}}^{\text{2}}\\ & ={\left(1.96\right)}^{2}\times 0.5\times (1-0.5)/{\left(0.07\right)}^{\text{2}}\\ & =196 \end{array}$$

Where,

Z = 1.96 for confidence interval at 95%
p = prevalence, 50%
q = 1-p
e = margin of error, 7%

Collected data were cross checked every day to ensure all the questions have been filled. All the collected data were entered into Microsoft excel and exported to SPSS version 22 for analysis. Simple frequency tables, cross tables and mean tables have been used to analyze data related to the study. Characteristics of the sample were categorized using mean and standard deviation.

## RESULTS

The mean age was 20.13±1.19 years for the respondents. The age of the respondents was categorized on the basis of mean age 20. The finding revealed that nearly two-third 132 (62.56%) of the students were less than equal to 20 years. Two-third of respondents 140 (66.35%) resides in rural municipalities. Caste/Ethnicity was categorized as per Nepal caste and ethnic groups with regional divisions and social groups (Table 1).

**Table 1:** Distribution of socio-demographic characteristics of study.

General characteristics	n (%)
Age	
≤ 20 years	132 (62.56)
> 20 years	79 (37.44)
Mean age ±SD	20.13±1.19
Gender	
Male	104 (49.26)
Female	107 (50.71)
Residence	
Urban (Sub-metropolitan city and Municipalities)	71 (33.65)
Rural (Rural Municipalities)	140 (66.35)

Regarding online classes nearly one-fourth of the respondents were not enjoying the class as per regular lecture class. Nearly two third 135 (63.98%) of the respondents felt online classes was not equally competent as regular lectures. A large number 178 (84.36%) of students found a great variation of online teaching from teacher to teacher (Table 2).

**Table 2:** Students experience during online classes.

General characteristics	n (%)
Enjoying online classes	
Not Enjoying	50 (23.70)
Enjoying in some extent	156 (73.93)
Enjoying fully	5 (2.37)
Students' opinion about online class	
Equally competent as regular class	32 (15.17)
Not equally competent	135 (63.98)
Can't say	44 (20.85)
Variation of Online teaching from teacher to teacher	
Yes	178 (84.36)
No	12 (5.69)
Can't Say	21 (9.95)

Most 208 (98.58%) of the medical and dental students had taken online class for the 1^st^ time. More than half 113 (53.55%) of the respondents used mobiles. One-third 71 (33.65%) of the students used mobile data to take the classes. Universal College of Medical Sciences and Teaching Hospital online classes were scheduled as 4-5 hours for 1^st^ year MBBS and BDS whereas 2-3 hours for MBBS and BDS 2^nd^ year. Based on average timing of the college, timing was categorized as less than equal to 4 hours and greater than 4 hours. The findings revealed that nearly two-third of the respondents had taken less than and equal to 4 hours online classes (Table 3).

**Table 3:** Status of online classes.

General characteristics	n (%)
Prior online classes taken	
No	208 (98.58)
Yes	3 (1.42)
Gadgets used for online Class	
Laptop	92 (43.60)
Mobile	113 (53.55)
Tablet/I-pad	4 (1.90)
Desktop	2 (0.95)
Internet Service used	
Wifi	140 (66.35)
Mobile data	71 (33.65)
Hours of online class taken per day	
≤ 4 hours	134 (63.51)
> 4 hours	77 (36.49)
Mean hour±SD	4.05±1.15

Nearly four-fifth 168 (79.62%) of the respondents faced internet disturbance during online classes. Similarly more than half 112 (53.08%) of the respondents faced mobile network problem, whereas little 26 (12.32%) had headache and eye strain due to online classes. Nearly half 112 (53.08%) of the students felt economic burden during online class (Table 4).

**Table 4:** Obstacles and constraints during online classes.

General characteristics	n (%)
**Difficulties faced during online class**	
Network problem (telecom tower)	112 (53.08)
Electricity problem	47 (22.28)
Headache	26 (12.32)
Eye Strain	26 (12.32)
Internet disturbance	168 (79.62)
Felt economic burden due to online class	
Yes	112 (53.08)
No	55 (26.07)
Can't Say	44 (20.85)

Fifty six (41.48%) of the students recommended to reduce the number of classes in online teaching, whereas few 20 (14.82%) provided constructive feedback for audio visual aids. Majority 199 (94.31%) of the respondents suggested to reduce the classes duration (Table 5).

**Table 5:** Students' recommendation for making online classes effective.

General characteristics	n (%)
Recommendation for competent class (n=135)	
Reduction in number of classes	56 (41.48)
Reduction in time for class	17 (12.59)
Focus in two way interaction	42 (31.11)
Provide audio-visual materials	20 (14.82)
Recommended technique for making class effective (n= 86)	
Focus on it advancement	52 (60.47)
Andragogy teaching style should be used	34 (39.53)
Students' recommendation for time duration	
≤ 3 hours per day	199 (94.31)
> 3 hours per days	12 (5.69)
Mean recommended time±SD	2.80±0.56

## DISCUSSION

This study assessed the perceptions of medical students towards E-learning. In our study, majority of students enjoying E-learning only to some extent (73.9%), fully enjoying E-learning were just five (2.4%), and not enjoying 50 (23.7%). This study is consistent with the finding of another conducted in Oman and UAE, where some students suggested that they would prefer the traditional method too as they can get bored in a virtual environment as well as no ease with use of technology.^7^

Our study indicates that out of 211students, 53.6% used mobile phones, 43.6% used laptops for online classes this is similar to Briz-Ponce et al study, which looked into in to mobile usage for learning among medical students of Coimbra University, and a strong attitude towards mobile use for learning (57%) and willingness to recommend it (40.5%) was noted.^8^

As regards the perception of students towards online class in comparison to regular class room teaching, majority of students felt online class is not equally effective 64%, and 15.1% students felt equally effective, and 33.1% could not say. A total of 86 (40.8%) students felt that regular class room teaching can't be substituted by online class, while 26.1% felt can be substituted and 33.1% students couldn't say. Majority of the students preferred traditional face to face class room teaching over online classes. This finding is quite different from a study of Solimann et al which was conducted at the College of Medicine at King Saud University (Saudi Arabia) had noted that the general perceptions of the students 63.5% were positive that an e-learning enabled training would enhance their learning process.^9^

In our study, majority of students had disturbance during online classes as Internet disturbance 79.6%, and electricity problem 22.3%. Similarly, many students 73.5% felt external disturbance during online classes. This finding is in consistent with the study by Subedi S, where more than half of the students 63.2% got disturbed for their online class because of electricity problem, and 63.6% because of internet problem.^10^

An alarming response that we received from this study was student's response to headache and eye strain during online classes which was 25% directing towards lockdown myopia.^11^

The sample of our study was taken only from one college, so the findings cannot be generalized to other medical colleges. The reliability of our data is entirely based upon the correct reporting of the participants. So, call bias might occur. Furthermore, an in-depth qualitative study is necessary.

## CONCLUSIONS

Most of students enjoyed the online classes to some extent where as understanding the subject content was moderate. Most of the respondents suffered from disturbances during online classes probably due to internet and electricity problem. When it is compulsory to conduct online classes, students felt that not more than three online classes per day should be conducted to avoid eye strain and headache.
